# Is biofilm formation intrinsic to the origin of life?

**DOI:** 10.1111/1462-2920.16179

**Published:** 2022-09-07

**Authors:** Ute Römling

**Affiliations:** ^1^ Department of Microbiology Tumor and Cell Biology, Karolinska Institutet Stockholm Sweden

## Abstract

Biofilms are multicellular, often surface‐associated, communities of autonomous cells. Their formation is the natural mode of growth of up to 80% of microorganisms living on this planet. Biofilms refractory towards antimicrobial agents and the actions of the immune system due to their tolerance against multiple environmental stresses. But how did biofilm formation arise? Here, I argue that the biofilm lifestyle has its foundation already in the fundamental, surface‐triggered chemical reactions and energy preserving mechanisms that enabled the development of life on earth. Subsequently, prototypical biofilm formation has evolved and diversified concomitantly in composition, cell morphology and regulation with the expansion of prokaryotic organisms and their radiation by occupation of diverse ecological niches. This ancient origin of biofilm formation thus mirrors the harnessing environmental conditions that have been the rule rather than the exception in microbial life. The subsequent emergence of the association of microbes, including recent human pathogens, with higher organisms can be considered as the entry into a nutritional and largely stress‐protecting heaven. Nevertheless, basic mechanisms of biofilm formation have surprisingly been conserved and refunctionalized to promote sustained survival in new environments.

## INTRODUCTION

Global bacterial infections, such as medieval plague caused by *Yersinia pestis*, pandemic cholera caused by *Vibrio cholerae*, whooping cough caused by *Bordetella pertussis* and tuberculosis caused by *Mycobacterium tuberculosis* contributed to our anthropocentric view of microbes acting as single‐cell planktonic organisms. Upon closer inspection of the disease process, however, these and other infectious agents and microbes are, rarely, if at all, observed as single planktonic cells. Multicellular biofilm‐forming microbes, which display as surface, interface or self‐attached cell aggregates, consisting of autonomous cells of diverse phylogenetic origin constitute the majority of microbial life. The success of this multicellular mode of growth during earth time is reflected by the fact that up to 80% of human bacterial infections are biofilm‐associated according to the National Institutes of Health (Flemming & Wuertz, 2019). Thereby, biofilm formation might be the major virulence factor of otherwise more benign microorganisms or play a role only in a spatially, temporally or functionally restricted part of the infection process. Be it in marine sediments, in the continental subsurface or in association with higher organisms, such as plants, invertebrates and humans, biofilm‐forming organisms are the predominant life form and drive to 100% geochemical processes (Flemming & Wuertz, 2019). The formation of biofilms, which were defined by Bill Costerton as ‘microbes adhering to each other and/or to surfaces or interfaces with the aid of a self‐produced (or environmentally based) extracellular matrix’ (Costerton et al., 1995), includes initial adherence, the assembly of microcolonies and monolayers of cells on surfaces. These initial structures transition into multicellular assemblies of differentiated self‐autonomous cells with tissue‐like properties by conducting developmental cycle(s) sophisticated genetically and environmentally programmed. The life cycle they enter switches reversibly between multicellularity and the, often motile, single‐cell planktonic state. Directed is this life style transition by the integration of a multitude of environmental and intrinsic signals on various regulatory levels (Asally et al., 2012; Chou et al., 2022; Palmer & White, 1997; Simm et al., 2004; Simm et al., 2014). Rather the rule than the exception, more than one distinct pathway leading to biofilm formation can be encoded by one microbial genome. Thereby, the genetically programmed biofilm formation which is highly flexible with respect to the contributing components is build up modularly from individual biofilm pathways and biosynthetic entities (Bundalovic‐Torma et al., 2020; Erskine et al., 2018; Korea et al., 2010; Low & Howell, 2018; Römling & Galperin, 2015; Zapotoczna et al., 2016). For example, distinct biofilms can be built by three different exopolysaccharides in *Pseudomonas aeruginosa*. Considering this flexibility, the genetic plasticity of biofilm genes and their lateral transfer, it might be challenging to develop strategies to tackle biofilm‐associated infections, although commonly acting activators of biofilm formation such as the ubiquitous second messenger cyclic di‐GMP have been identified (Hee et al., 2020; Römling et al., 2013; Trampari et al., 2021). Refractoriness to stress conditions might be founded already in the origin of biofilm formation, which is proposed to be intimately coupled to the origin of life itself. To lay out the fundamentals of ancient metabolisms which are preserved until today even in evolved organisms might also aid in the tackling of chronic infections (Falkowski et al., 2008; Greening et al., 2022).

## EVOLUTION OF LIFE IN CLOSE CONTACT TO SURFACES

**FIGURE 1:**
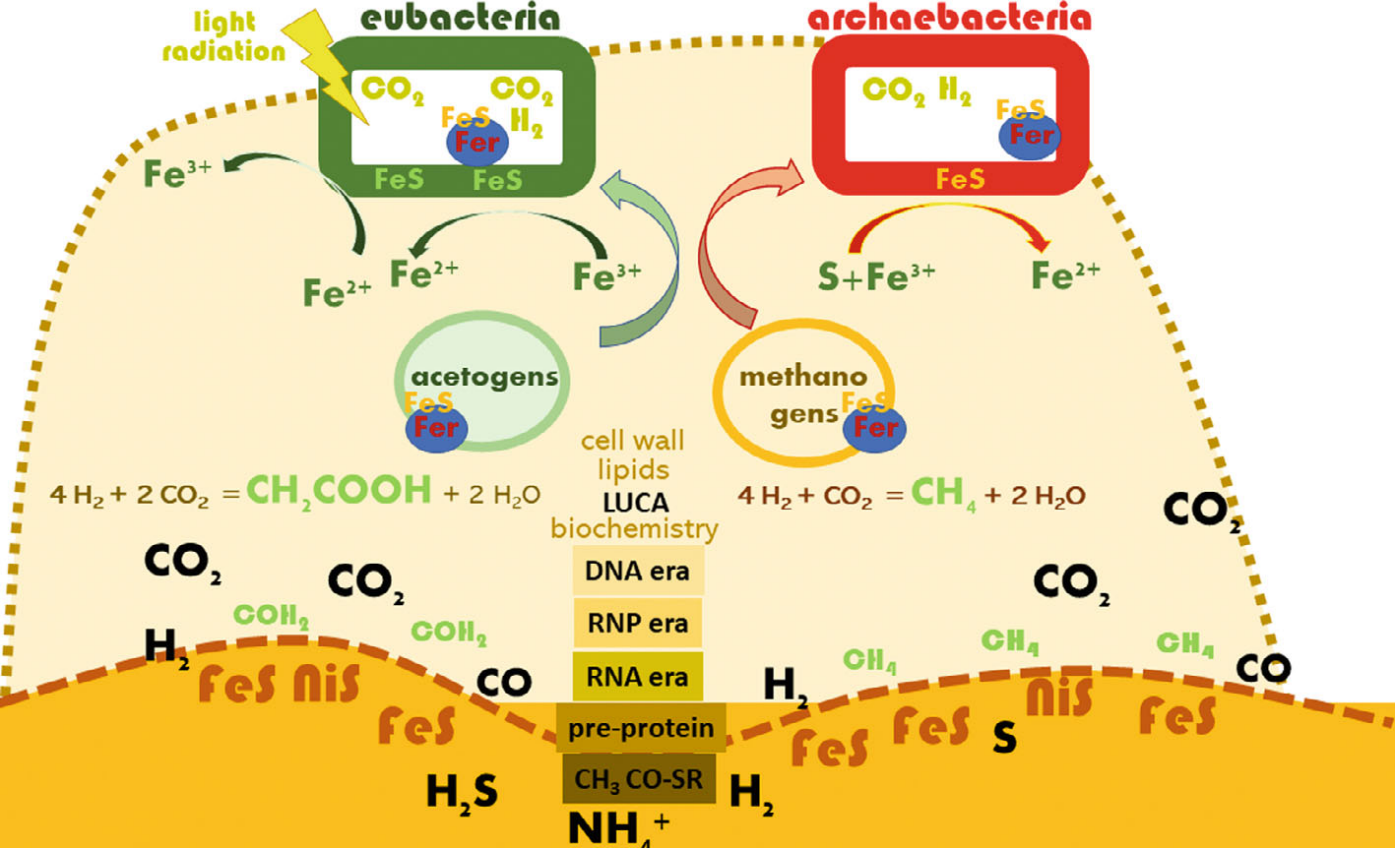
The biofilm mode of life has its origin in the development of life on catalytic surfaces (minerals) such as FeS, FeNiS or FeS/SiO_2_ clusters that are intimately associated with its emergence. On these and/or similar redox‐active clusters in the presence of molecules such as hydrogen (H_2_); carbon dioxide (CO_2_); carbon monoxide (CO); ammonium, (NH_4_^+^); and hydrogen sulfide (H_2_S); created by geochemical reactions such as serpentinization, the first organic molecules including formamide, acetate and methane and high‐energy acetyl‐thioesters arose. Subsequently, upon the consecutive development of more complex organic molecules, the RNA, RNP (ribonucleoprotein) and DNA era, perhaps preceeded by pre‐proteins with metal cofactors similar to the ancient ferredoxin fold emerged. The first biological membranes (organized around semipermeable energized FeS or FeS/SiO_2_ walls with built up ion gradient) and cell walls in protected compartments and a last universal common ancestor (LUCA) were similarly facilitated in contact with FeS and/or similar redox‐active mineral clusters resulting in first primodal biotic energy metabolisms (Jordan et al., 2021). Evolution would have eventually led to the incorporation of FeS clusters into respiration‐associated proteins with the ferredoxin fold (Fer) central to the origin of ancient redox metabolism, as still universally found in all domains of life (Raanan et al., 2020). Of note, though, the third domain Eukarya synthesizes Fe‐S protein only in mitochondria and plastids (Braymer et al., 2021). Suggested primeval (prokaryotic) forms of life, with an acetogenic and methanogenic life form on mineral surfaces would have been sessile, essentially constituting ancestral biofilms of the eubacterial and archaeabacterial line. Indeed, methanogens were observed to form mono‐species biofilms in today's hypothermal vents (Brazelton et al., 2011). Respiration occurred with subsequent transformation of minerals using ferrous and ferric iron and sulfur and sulfur molecules in different redox stages as most ancient redox pairs. Over time, diversification of cellular life has led to populations of cells specialized on biofilm or planktonic lifestyles dependent on the availability of redox pairs and possibilities for electrohomeostasis. In the presence of respiratory electron acceptors, some bacterial species or strains take over the niche from competitors through (planktonic) population increase, thereby taking prevalent control of the metabolic resources available at the site. Inspired by Sousa et al. (2013).

After the birth of our solar system and the proto‐earth 4.6 and 4.5 billion years ago, respectively, life is believed to have originated approximately 3.8 billion years ago. Biofilm‐forming organisms have been traced back as long as 3.5–3.8 billion years (Mojzsis et al., 1996; Schopf, 1993). Named as stromatholites in the case of cyanobacteria or more general microbialites, these fossils are observed as surface‐spread monolayers of microbial consortia blended with inorganic sediments, which were explicitly described as early as 1883 (Awramik & Sprinkle, 1999; Shapiro, 2007). Here, I argue that biofilm formation defined in the original sense as the close association of microbial cells with surfaces is in fact intrinsic to the prerequisites and molecular mechanisms that enabled the emergence of life (Figure 1).

The emergence of life including the metabolism and energy conservation as well as cellular organization required solid, catalytically competent surfaces (Figure 1). Thus far before dividing into two structurally fundamental different branches of early life, the eubacterial and archaebacterial protocells, the last universal common ancestor (LUCA) emerged from inorganic template predecessors not only in intimate surface‐association or even being embedded within (Baross & Martin, 2015; Koonin, 2003; Sousa et al., 2013; Stoodley et al., 2002; Trevors, 2011; Wachtershauser, 1992), but with the active contribution of reactive inorganic surfaces to the development of metabolism and as a cell template. Which of various environmental conditions were most beneficial for the emergence of life and thus can be regarded as likely origin is still debated (Barge, 2018; Camprubi et al., 2019; Ebisuzaki & Maruyamab, 2017; Walker et al., 2017). Among the scenarios are conditions similar to contemporary alkaline hydrothermal vents, which are considered to provide appropriate prerequisites for the cradle of life such as chemical disequilibria, natural gradients and transition metals at different redox states for initial catalysis (Lane & Martin, 2012; Maruyama et al., 2019; Russell et al., 1993). With its foundation in fundamental geochemical processes such as serpentinization, the transformation of minerals and water, the production and accumulation of inorganic molecules provided the basis for the transition to biochemical processes and the emergence of organic molecules (Amenabar & Boyd, 2019). Catalytic, electron‐donating and/or accepting iron/nickel sulfur clusters subsequently enabled the acceleration (relative to Earth time) of chemical reactions with inorganic gases CO, CO_2_, H_2_ and NH_3_ as substrates to provide the first organic molecules such as formate, methane, acetic acid, lactate, pyruvate and subsequently even amino acids. Indeed, these ancient inorganic and organic molecules or variants of them are even today central to carbon fixation and energy metabolism (Gong et al., 2016; Greening et al., 2022; Huber & Wächtershäuser, 1998; Kessler et al., 2019). Furthermore, included in sediments, this type of environment led to the concentration of small molecular compounds and limited the diffusion of molecules (Sousa et al., 2013).

Energy conservation is one of the prerequisites of life. In the primeval Earth before the onset of life. the energy‐conserving reactions have been initially facilitated through catalysis of the redox reactions on iron‐sulfur mineral walls or mineral walls of similar transition metal composition, perhaps mixed with silica. Conservation of energy in chemical form as a natrium or proton gradient occured over those semi‐permeable inorganic walls (Lane & Martin, 2012; Sousa et al., 2013). The chemical conservation of energy has been first demonstrated abiotically on energized semipermeable FeS walls or semi‐permeable membranes, which enabled synthesis of simple organic molecules, such as high‐energy acetyl‐thioesters (Dibrova et al., 2015). These types of energy conservation were subsequently been replaced by substrate‐level phosphorylation and chemiosmotic energy conserving mechanisms as lipid bilayer membranes evolved. Chemical energy conservation is coupled to the synthesis of ATP as a universal energy currency, driven by the built‐up of ion, Na^+^, and/or H^+^ gradients, over a nearly impermeable membrane. Such a biogenic membrane allows transport against a concentration gradient, uphill redox reactions and fundamental energy‐requiring processes such as cell division and motility (Benarroch & Asally, 2020). In the primeval Earth before the onset of life, these energy‐conserving reactions have been initially facilitated through catalysis of the redox reactions on iron–sulfur mineral walls or mineral walls of similar transition metal composition and perhaps mixed with silica, with conservation of energy in chemical form as a proton gradient over those semi‐permeable inorganic walls (Lane & Martin, 2012; Sousa et al., 2013). These principles of bioenergetics are valid even today in organisms with rudimentary mechanisms of energy conservation (Kracke et al., 2015). As such many organisms obtain the energy for life in the form of the ubiquitous currency ATP by fermentation, substrate‐level phosphorylation of energetically high molecules such as acetyl‐phosphate or conserve energy over membranes without the sophisticated respiratory chains widespread today among bacteria (Unden & Bongaerts, 1997). Nevertheless, the first cells almost certainly developed slowly even relative to earth time; likewise, today's microbes in energy‐poor subseafloor sediments can have an estimated doubling time of up to 1000 years (Braun et al., ; Trembath‐Reichert et al., 2017). Subsequently, the acquisition of energy was optimized (Pfeiffer et al., 2001), which perhaps already early needed to be traded against the attack of antimicrobials and phages (Benarroch & Asally, 2020; Kalasauskaite & Grinius, 1979; Labedan & Goldberg, 1979) as those require a membrane‐potential to pass the cytoplasmic membrane.

The emergence of respiration, accompanied further by oxidation of carbon sources to CO_2_ resulted in better energy conservation. Energy from electron flow was used to build up sodium and proton gradients by spatially separated energetically favoured redox reactions (Pfeiffer et al., 2001). These spatially separated redox reactions require membrane diffusible lipid soluble redox compounds such as quinones and methanophenazine and membrane‐associated proteins containing iron–sulfur clusters, flavins and heme‐containing iron, with ferredoxin and cytochrome c, respectively, as the most ancient protostructures (Abken et al., 1998; Kotloski & Gralnick, 2013; Raanan et al., 2018; Raanan et al., 2020; Russell et al., 1998). Respiration, which leads to a higher energy gain than fermentation, was originally anaerobic but upon the availability of oxygen as an electron acceptor, enabled aerobic metabolism. The use of oxygen as an electron acceptor significantly extended the range of available redox pairs, and enhanced energy gain and ecological opportunities (Brochier‐Armanet et al., 2009; Harrison et al., 2015; Unden & Bongaerts, 1997). Equally contributed the lateral transfer of respiratory modules (Clark et al., 2013; Deng et al., 2019; Kamal et al., 2021). With the conservation of energy in a chemiosmotic H^+^ or Na^+^ membrane gradient, chemical energy in the form of ATP is ubiquitously gained by conceptually similar, but structurally distinct ATP synthases (Deckers‐Hebestreit & Altendorf, 1996; Gruber et al., 2014). However, there are indications that even today fermentation can be coupled to extracellular electron transfer which promotes more rapid proliferation and metabolism indicating that intrinsic redox reactions feedback on metabolism (Tejedor‐Sanz et al., 2022). Similarly to the origin of simple central biomolecules and its energy conservation, emergence of other cellular components such as lipids are supposed to have required catalysis on autocatalytic surfaces (Bernal, 1949; Wächtershauser, 1988).

**FIGURE 2:**
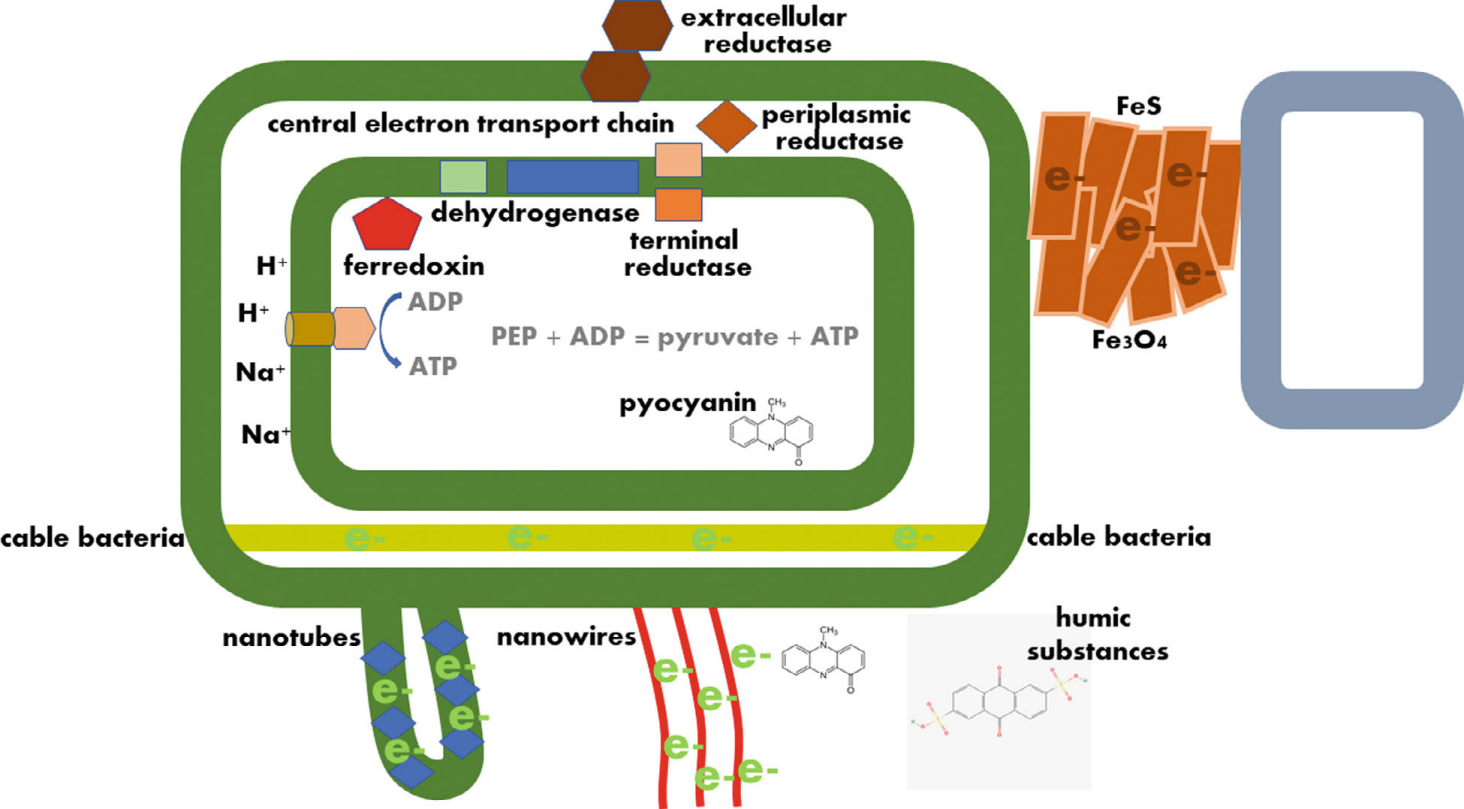
Different modes of electron transfer for energy gain. In respiration, an electron transport chain located predominantly in the cytoplasmic membrane consisting of FeS cluster‐containing proteins, flavin and heme‐containing integral membrane proteins and membrane diffusible quinone‐based redox compounds transfers electrons along the redox gradient with the subsequent built up of an ion gradient, H^+^ or Na^+^, over the membrane for the production of the energy equivalent ATP. Methyl transfer is an alternative reaction to built up an ion gradient. Substrate‐level phosphorylation is an alternative way to general ATP. The input into the central electron transport chain comes from different metabolic processes such as the catabolism of sugars, energy and carbon sources and nucleotide biosynthesis performed by oxidoreductases; the most prominent the NAD(P)H/NAD^+^ dehydrogenase complex, the succinate and formate dehydrogenase complex, hydrogenases, but also the dihydroorotate dehydrogenase in the nucleogenesis pathway and alternative oxidoreductases (Kaila & Wikstrom, 2021). The periplasmic located CymA reductase of *Shewanella oneidensis*, a homologue of NapC in *E. coli*, has been shown to respire ferric iron not only in its ingenious host, but also in *E. coli* (Gescher et al., 2008). Other means to transport electrons along the redox gradient are conductive minerals such as iron sulfide or iron oxide (electric synthropy), intercellular nanotubes, nanowires (such as conductive type IV pili in eubacteria or archaella in archaebacteria), intrinsic and extrinsic diffusible redox active substances such as pyocyanin and the humic model compound anthraquinone‐2,6‐disulfonate, respectively, as well as an periplasmic electron transfer in cable pili. PEP, phosphoenolpyruvate

Extending this scenario, primitive forms of cellular life can have evolved as inherently sessile surface‐associated life forms in direct contact with minerals or within sediments. Indeed, also other fundamental metabolic processes such as anoxygenic photosynthesis thought to represent one of the earliest forms of metabolism and an alternative mode of energy conservation using light as the external energy sources is based on the oxidation of Fe^2+^ to Fe^3+^ (Ozaki et al., 2019; Xiong et al., 2000). Furthermore, Fe^3+^ has been recognized as the most ancient and a central electron acceptor in respiration (Vargas et al., 1998) and Fe^2+^ is in a reverse reaction also used as an electron donor (Burini et al., 2018). Moreover, biofilms form even today on metal sulfides and sulfur, the second central redox‐active compound enabling ancient metabolism. Thus, surface association enabled various modes of energy conservation with subsequent development of a protocell and its growth connecting biofilm formation intimately to the origin of life. The early type of surface associated biofilm formation has been evolutionary maintained until today in microbes with microbes still respiring extensively using different iron and sulfur redox stages in minerals (Byrne et al., 2015; Kato et al., 2012; Liu et al., 2013). The harsh conditions upon the emergence of life also put forward the hypothesis that resource‐restricted damaging‐exposed environmental conditions, commonly called stress, has been the rule rather than the exception as experienced by microbes today (Merino et al., 2019). Thus, elevated repair and persistence mechanisms, which are a hallmark of today's biofilms, were necessary to evolve concomitantly with the emergence of microbial (biofilm) life (Fernandez et al., 2018). Biofilm formation and the physiology of microbes has, however, evolved and diversified with the radiation of the two fundamentally different prokaryotic forms of life, the eubacteria and the archaebacteria. On the phylogenetic level, collectively, those organisms have access to all thermodynamically possible redox couples in minerals and in soluble form. The introduction of oxygen as an electron acceptors during the Great Oxygenation Event widened further the spectrum of potential redox pairs. Regulation of those processes occurs by the presence and the concentration of the terminal electron acceptor which determines the expression of the terminal reductase (Unden & Bongaerts, 1997). For example, in *Escherichia coli*, there exist at least 10 terminal reductases. These integral membrane or periplasmic proteins, dedicated for the respiration of substrates such as oxygen, dimethylsulfoxide (DMSO), trimethylamine‐*N*‐oxide (TMAO), fumarate, nitrate and nitrite are expressed upon substrate availability. Reductases can, however, also be located in, or in association with, the outer membrane with the catalytic centre facing the exterior not only upon reduction of solid substrates (Figure 2). Reduction of ferric ions by *E. coli* NapC, an integral member of the respiratory chain which transfers electrons to the periplasmic NapAB nitrate reductase indicates ancient functionality, but also further refunctionalization of members of the respiratory chain (Gescher et al., 2008).

The most ancient kinds of metabolism, as found in acetogens, in which carbon fixation via CO_2_ is subsequently reduced by H_2_ to acetate, and methanogens, in which fixation of CO_2_ is subsequently reduced by H_2_ to methane (Figure 1), possess neither heme nor quinones and hold only rudimentary respiratory chains. Nevertheless, built up of chemically stored energy requires ion pumps, membranes, protein‐framed iron–sulfur clusters such as in ferredoxin, an ion complexation central to the origin of ancient redox metabolism (Raanan et al., 2020), to facilitate redox reactions (Dibrova et al., 2015; Rosenbaum & Müller, 2021; Skulachev, 1984). Despite using volatile redox pairs for energy and carbon acquisition, mono‐species biofilm formation of methanogens occurs in hyperthermal vents (Brazelton et al., 2011). Thereby, energy provided by light or radiation equally as electron bifurcation, the coupling of energetically favourable with unfavourable redox reactions, can enable carbon fixation and energy storage (Buckel & Thauer, 2018; Peters et al., 2018). Eventually, energy storage evolved towards quinone‐ or methanophenazine‐based proton gradient generation using heme‐containing cytochromes in respiratory chains including their regulatory components (Aussel et al., 2014; Kroger & Dadak, 1969; Nitzschke & Bettenbrock, 2018). In fact, the fundamental origin of the components of the respiratory chain is reflected by their combinatorial assembly using a redox protein construction kit (Baymann et al., 2003).

## PLANKTONIC CELLS EVOLVED SECONDARY TO SURFACE‐ASSOCIATED CELLS

What does the hypothesis of the ‘emergence of life as a biofilm’ imply? Accordingly, and most importantly, planktonic cells must have evolved secondary to surface attached cells. Prerequisites that enabled the development of planktonic cells include the availability of electron donors and acceptors in solution (and subsequently on biotic surfaces) combined with high‐affinity terminal reductases.

Biofilm‐forming microbes with their intimate surface association have developed various modes of direct and indirect electron transfer to interact with abiotic surfaces and, to communicate through abiotic electron transfer, via conductive minerals, with each other (Dong et al., 2020; Figure 2). Cells can deliver electrons via nanotubes, membrane extrusions with nanotubes and nanowires, periplasmic structures, proteinaceous type IV pili or archaella appendages to abiotic surfaces and/or even, today, in biotechnological applications, for current generation to conductive metals (Dubey & Ben‐Yehuda, 2011; Lovley & Holmes, 2020; Nealson & Finkel, 2011; Reguera et al., 2005). Furthermore, diffusible small redox active molecules emerge either synthesized endogenously and/or are provided exogenously from the environment to contribute efficiently to extracellular electron transfer. Examples are the antibiotic pyocyanin and the humic model compound anthraquinone‐2,6‐disulfonate, respectively (Hernandez & Newman, 2001). The biological significance of extracellular electron transfer includes the secretion of small molecular metabolic waste products that can be catabolically used by other microorganisms as a carbon source and thus contribute to the optimal use of energized electrons and energetically higher molecules for energy conservation, resulting in a macroorganism, for example, in cell dense sediments.

**FIGURE 3:**
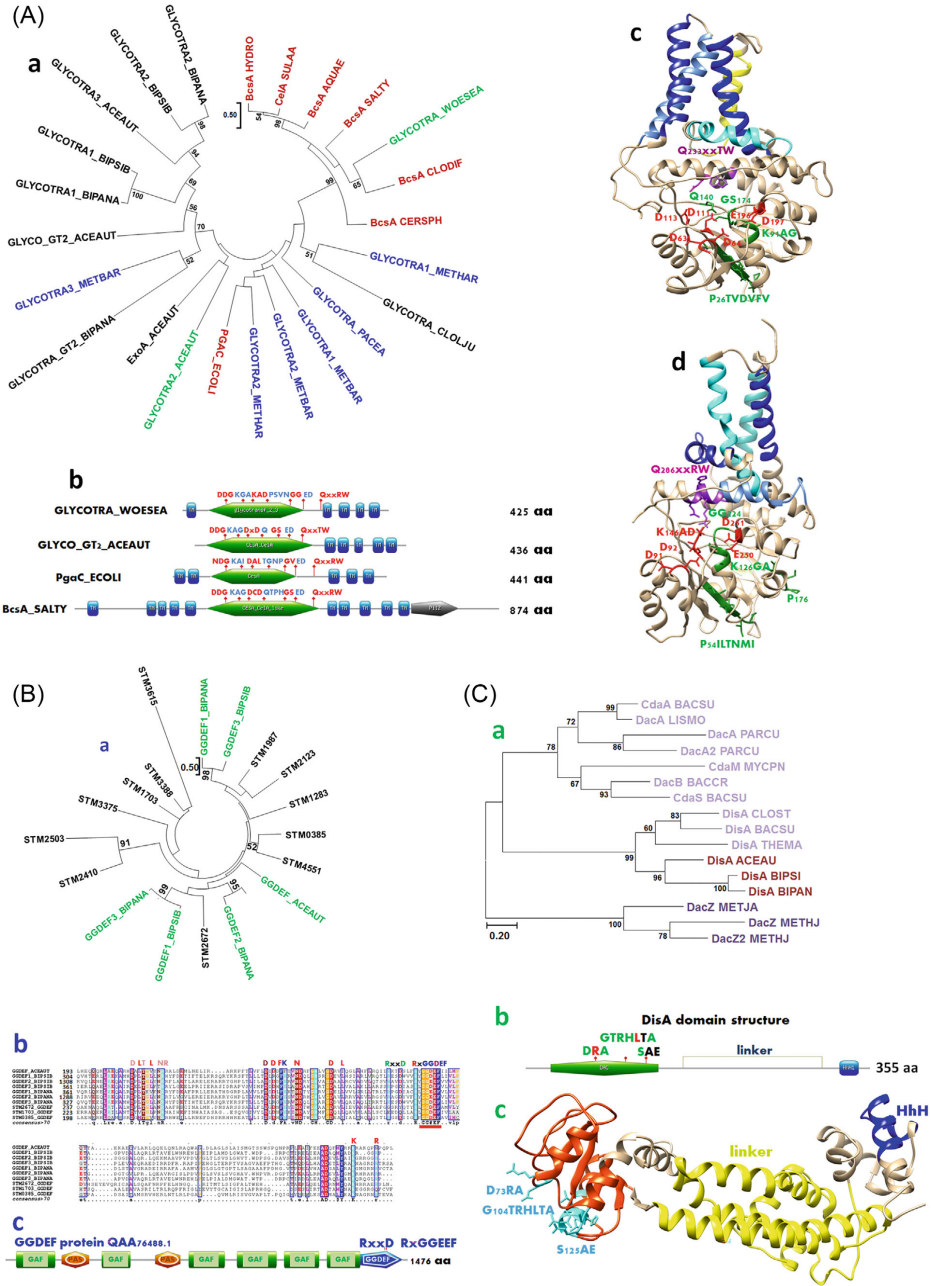
Presence of biofilm gene products in deeply branching bacteria and/or archaea indicate an ancient origin of molecular mechanisms of biofilm formation. (A) Poly‐1,6‐beta‐*N*‐acetylglucosamine/cellulose/alginate synthase genes have been predicted to be present in LUCA of bacteria and archaea (Weiss et al., 2016). (a) Phylogenetic position of the core catalytic domain of representative glycosyltransferases identified by Blast searching (Altschul et al., 1990), archaeal and bacterial deeply branching phyla with the poly‐beta‐1,6‐*N*‐acetyl‐glucoseamine synthase PgaC of *Escherichia coli* and the cellulose synthase BcsA of *Aquifex aeolicus*. The three (nearly) complete genomes of Candidatus *Acetothermum autotrophicum* (ACEAUT), Candidatus *Bipolaricaulis sibiricus* Ch78 (BIPSIB) and Candidatus *Bipolaricaulis anaerobius* Ran1 (BIPANA) and one identified homologous glycosyltransferase each from DPANN archaea and *Methanosarcina* were also included in the phylogenetic analysis. Red, domains from reference proteins; green, structural models in (c) and (d). (b) Domain structure and glycosyltransferase 2 (GT2) characteristic motifs and (c) structural models of an archaeal (MBP4151113.1, Woesearchaeota archaeon) and a Bipolaricaulota representative (BAL58984.1, C. *A. autotrophicum*). The structural models were constructed with Phyre2 (Kelley et al., 2015). In blue and yellow, transmembrane helices as predicted by TMHMM 2.0 (Krogh et al., 2001). The GT2 domains are named according to the domain with lowest expect values. (B) Phylogenetic position of GGDEF domains of GGDEF domain proteins of representative Bipolaricaulota species. (a) Blast searching (Altschul et al., 1990) the three (nearly) complete genomes of C. *A. autotrophicum*, C. *B. sibiricus* and C. *B. anaerobius* with the diguanylate cyclase AdrA of *Escherichia coli* recognized one, three and four GGDEF domain proteins (green). Reference GGDEF domain proteins of *Salmonella typhimurium* are described (Römling, 2020). (b) Alignment of GGDEF domains of GGDEF domain proteins of C. *A. autotrophicum*, C. *B. sibiricus* and C. *B. anaerobius* with selected GGDEF domains of *S. typhimurium* proteins. Underlined in red is the conserved catalytic GGDEF motif. (c) Domain structure and conserved residues of the GGDEF domain protein QAA76488.1 of C. *B. sibiricus* from the Bipolaricaulota phylum. (C) Diadenylate cyclase proteins of representative Bipolaricaulota species, C. *A. autotrophicum*, C. *B. sibiricus* and C. *B. anaerobius* as identified by Blast searching with the diadenylate cyclase DisA of *T. maritima*. (a) Phylogenetic position of the diadenylate cyclase (DAC) domain of DisA proteins from C. *A. autotrophicum* (BAL58991.1), C. *B. sibiricus* (QAA77389.1) and C. *B. anaerobius* (WP_122030516.1) in relation to the DAC domain of representative proteins of the distinct and characterized diadenylate cyclase groups DisA, CdaA (DacA), CdaM, CdaS (DacB), CdaZ (DacZ) (Römling, 2008; Witte et al., 2008). (b) All currently identified diadenylate cyclase proteins of Bipolaricaulota species belong to the DisA group with an identical domain structure of DAC‐linker‐(helix‐hairpin‐helix) non‐specific DNA binding motif class 1 as exemplified by the DisA protein of C. *A. autotrophicum*. Catalytic residues are partially conserved in DisA of C. *A. authtrophicum* with the DGA motif changed to DRA, the RHR motif in the context of GTRHRxA being GTRHLTA and the catalytic serine conserved in the context of SAE. DisA proteins of C. *B. sibiricus* and C. *B. anaerobius* have the RHR motif conserved. (c) Structural model of the DisA protein of C. *A. autotrophicum* constructed with Phyre2 (Kelley et al., 2015) and displayed in Chimera (Pettersen et al., 2004). Protein designations in Supplementary Material S1

Thus, the surface‐associated protocells might have been naked cells emerging from a prebiotic gel (Trevors, 2011) without an extracellular matrix surrounding them. This aspect would be distinct from the hallmark characteristic for todays' biofilms (Costerton et al., 1995). It cannot be excluded though that a primordial musilace existed (Russell et al., 1993; Shapiro, 2007) or extracellularly secreted DNA, an extracellular component of biofilms even today (Whitchurch et al., 2002), might have already early served as a biofilm extracellular matrix component. This is not unlikely considering the higher permeability of early membranes (Dibrova et al., 2015), as a threshold in permeability is a prerequisite for secretion (DeFrancesco et al., 2017). So why and how did extracellular matrix production of surface attached biofilm cells evolve. While the ‘how’ cannot be answered directly, the production of an extracellular matrix certainly has provided an additional layer of protection. More diversified metabolic pathways emerged in surface‐attached cells or their cellular predecessors might have required protection from radiation, fast or uncontrollable redox reactions or interfering metal ions. Indications for an early arisal of a polysaccharide‐based extracellular matrix are a poly‐1,6‐beta‐N‐acetylglucosamine/cellulose synthase like glycosyltransferase family 2 protein encoding genes among the genes most likely present in the last universal common ancestor of bacteria and archaea (Weiss et al., 2016). Those gene products encoded in today's genomes by members of deepest branching bacterial phyla such as Dictyoglomota, Coprothermobacterota, Caldiserica and Bipolaricaulota equally as in Archaea, although shorter in length, structurally resemble cellulose synthases in its core functional domain (Figure 3A and data not shown). Obviously, the advantages of extracellular matrix production were higher than the disadvantage; which are, for example, to be at a larger physical distance to the respective solid electron donors and acceptors. To overcome this hurdle, one can hypothesize that biofilm extracellular matrix components can associate with respective electron donors/acceptors for efficient energy gain (Keren‐Paz & Kolodkin‐Gal, 2020), as inorganic matter is integrated for stabilization of biofilm structures (Keren‐Paz & Kolodkin‐Gal, 2020). As another aspect, upon division of protocells attached to a surface, not all cells in multicellular structures have equal access to the redox pairs which has perhaps severely limited the proliferation of merely surface‐attached protocells. Production of matrix and other extracellular structures would thereby have enabled physical closeness with efficient transfer of electrons and small molecules and enabled an extension of the biofilms (Li et al., 2016; Nielsen et al., 2010).

Cellulose, a 1,4 beta‐glucan, which is solely composed of the most chemically inert sugar alpha‐d‐glucose, might have been one of the early extracellular polysaccharide matrix components. Indeed, cellulose is considered to serve as a biosignature in rock records as an indication of microbial life (Zaets et al., 2014) and is produced by microbes from deep braches of the phylogenetic tree of the phylum Aquificales, such as *Aquifex aeolicus* and *Hydrogenothermus*. What is again surprising though is the maintenance and modified reuse of such an ancient component, as cellulose production has been conserved in evolved human pathogens as in the gamma‐proteobacterium *S. typhimurium*, which forms cellulose‐based biofilms within immune cells to restrict virulence (Ahmad et al., 2016; Pontes et al., 2015; Zogaj et al., 2001). In metabolic agreement with an ancient origin of cellulose is the fact that UPD‐glucose for cellulose biosynthesis is provided by the gluconeogenesis pathway in *S. typhimurium* (White et al., 2010), an anabolic pathway, more ancient than catabolic glycolysis, for the supply of sugar molecules from acetate synthesized by the Wood–Ljungdahl pathway of carbon fixation from CO_2_ reduced by H_2_ (Say & Fuchs, 2010).

Preliminary bioinformatics analyses indicated also the presence of GGDEF domain proteins indicative of the ubiquitous biofilm activator, the second messenger molecule cyclic di‐GMP or, as synthesized by a restricted subset of GGDEF domains, cyclic AMP‐GMP (Hallberg et al., 2016). These proteins domains are of ancient origin as present in representatives of deepest branching bacterial phyla, such as Dictyoglomota, Coprothermobacterota, Caldiserica and Bipolaricaulota as well as in clostridial acetogens (Figure 3B and data not shown). Cyclic AMP‐GMP signalling presumably regulates adhesins and cytochromes, which are required for extracellular electron transfer to iron oxides in *Geobacter* (Hallberg et al., 2016; Nelson et al., 2015). Cyclic di‐GMP intercedes in the shift between the fundamental single‐cell life styles sessility and motility and can upregulate c‐type cytochromes involved in electron transfer in some species. Cyclic di‐GMP concurrently regulates other viable physiological pathways such as DNA repair and photosynthesis (Römling et al., 2013). The almost complete absence of GGDEF proteins in the archaeal lineage (presence of GGDEF domain protein need to be confirmed in archaeal contigs, unpublished data) is not an argument for the lack of cyclic di‐GMP signalling in a last ubiquitous common ancestor as this heat‐stable second messenger can rapidly disappear on evolutionary time scales even in free‐living bacterial genera without close association with a host (Liu et al., 2020; Nelson et al., 2015; Nelson & Breaker, 2017). Although today less abundant with respect to overall copy number of synthesizing and degrading gene products, cyclic di‐AMP can be the (or among) common second messenger signalling molecules as it signals DNA damage and surveys downstream repair mechanisms as well as regulates fundamental physiological processes such as potassium and osmohomeostasis (Corrigan & Grundling, 2013; Manikandan et al., 2018; Römling et al., 2013; Witte et al., 2008). Cyclic di‐AMP signalling affects biofilm formation by regulation of cell wall biosynthesis and eDNA secretion, which might be related to cell membrane/envelope permeability (DeFrancesco et al., 2017; Townsley et al., 2018). Importantly, genes for cyclic di‐AMP synthesizing enzymes are common to Archaea, deepest branching bacterial phyla such as Dictyoglomota, Caldiserica and Bipolaricaulota, present in model acetogen *Clostridium ljungdahlii* among other acetogens, other Gram‐positive bacteria, but rarely in Gram‐negative species (Figure 3C and data not shown; Yin et al., 2020). However, the low sequence conservation of GGDEF/DAC domains and their association with a diversity of signal receiver domains in these phyla indicates that those genes have diverged earlyand might have been refunctionalized (Moradali et al., 2022). Such signalling genes might not be recognized as most ancient vertically evolved genes in homology searches due to such early interdomain recombination events which placed the enzymatic domains under the post‐translational control of a diversity of intra‐ and extra‐cellular signal domains and due to lateral gene transfer which targets cyclic di‐GMP signalling more frequently than expected on average (Madsen et al., ; Moody et al., 2022; Römling, 2008; Weiss et al., 2016). Indeed, one can hypothesize that biofilm and respective regulatory genes must have been subject to extended early lateral gene transfer to adapt the capability to form a diversity of distinctly regulated biofilms adapted to respective environmental conditions. Furthermore, one might ask the question why in particular cyclic di‐AMP and cyclic di‐GMP had been selected to ubiquitously direct biofilm related physiology and metabolism. As an argument from a chemical perspective, in comparison to the monocyclic cyclic AMP and cyclic GMP, the covalent bonds in the di‐cyclic molecules experience less tension and thus lead to more stable molecules (Römling, 2012). On the other hand, a wide spectrum of diverse cyclic di‐nucleotides including cyclic AMP‐GMP, cyclic oligo‐nucleotides and even pyrimidine‐based cyclic nucleotides have recently been identified (Braun et al., 2021; Kazlauskiene et al., 2017; Morehouse et al., 2020). To what extent these and other nucleotide‐based signalling molecules play a role in physiology and metabolism of the majority of under‐investigated environmental microbes beyond phage defence systems remains to be determined.

## DIVERSIFICATION OF THE BIOFILM RESPONSE

As previously hypothesized in this opinion paper, biofilms originated concomitantly with life. The most fundamental behaviour towards ancient solid electron donors and acceptors and the cellular prerequisites for direct most effective energy gain from solid mineral electron acceptors (Harris et al., 2010; Harris et al., 2012) was therefore biofilm formation. With most ancient and most abundant iron and sulfur‐based respiration requiring solid surfaces, biofilm formation could be subsequently extended towards additional solid inorganic electron acceptors. Upon the availability of soluble inorganic and organic electron acceptors and other energy‐related signals, solid‐surface biofilm formation desisted to be mandatory for energy‐gain, but a multicellular microbal community in the form of cell aggregates (flocks) has still been a prerequisite for survival as even today, single planktonic microbial cells are rarely found in nature. With the presence of solid and soluble redox pairs, the biofilm response could subsequently further diversify. Thereby, modern free‐living microbes to access a diversity of solid electron acceptors and organic matter by motility combined with chemotaxis (Nealson et al., 1995). Motility opposite to sessility (biofilm formation) is not necessarily required to trigger initial biofilm dispersal. Higher organisms might have initially refunctionalized production of soluble organic electron acceptors such as TMAO and dimethylsulfoxide, wide‐spread in marine sediments and aquatic environments (Lee at al., 1999; Sun et al., 2018), respectively, to attract microorganisms. To occupy all the different ecological niches effectively, the respiratory responses among the microorganisms have diversified and can be quickly modulated. This is indicated by the fact that respiratory modules, signaling components and biofilm genes, are found on genomic islands and plasmid being subject to lateral gene transfer (Clark et al., 2013; Gomaa et al., 2021; Mangalea et al., 2017) Madsen at al., 2016. The responses towards certain electron acceptors may even have been reverted among the species. For example does nitrate respiration of the gastrointestinal pathogen *S. typhimurium* lead to reduced biofilm formation but triggers motility in order to promote invasion of the gut epithelial cell line and virulence, while in other bacterial species, nitrate respiration promotes biofilm formation (Martin‐Rodriguez et al., 2021; Miller et al., 2022; Rivera‐Chavez et al., 2016; Römling et al., 1998). Other respiration‐associated compounds such as NO can concentration dependent differentially regulate biofilm formation (Schmidt et al., 2004). Such a differentiated microbial behaviour can extend to the strain level to provide the opportunity of a wide niche occupation for individual strains within a species. As described above most possible ecological breadth within a species can thus be achieved through strain‐dependent biofilm‐responses towards electron acceptors, which allows differential occupation of ecological niches and most beneficial responses to diverse environments (Martin‐Rodriguez et al., 2021; Van Alst et al., 2007).

## DISCUSSION

The early origin of life spanning from the emergence of first organic molecules to fundamental energy and carbon fixation mechanisms in bacteria and archaea has been thoroughly studied. Development of alternative aspects of microbial physiology and metabolism such as the time line of origin, evolution and diversification of surface‐dependent energy conservation processes and the integration of redox‐active minerals into cell physiology has only been analysed at a rudimentary level. Equally, the time line of origin, evolution and diversification of biofilm formation including its task distribution and cell differentiation into, for example, persister cells, has not been performed. Identification of the ancient inventory and the deep phylogeny of biofilm genes including diversification of mechanisms and regulation of biofilm formation in the deepest branching bacterial and archaeal phyla can identify the most ancient processes. Equally addressing in detail the evolution of energy preserving modules with the analysis and integration of ancient energy‐gaining pathways such as anaerobic sulfur respiration (Hedderich et al., 1998) can give a first glimpse on these processes. On the other hand, while the presence of biofilm components such as the genetic modules for the production of the poly‐beta‐1,6‐*N*‐acetyl‐glucoseamine and cellulose exopolysaccharide and the contribution of eDNA to the extracellular matrix have been realized to occur in bacteria throughout the phylogenetic tree, its origin has not been defined (Mack et al., 1996; Whitfield & Howell, 2021; Zogaj et al., 2001). Equally, although a Gram‐negative acyl‐homoserine quorum sensing system has also been identified punctually in archaea (Zhang et al., 2012), a deep phylogenetic origin of biofilm‐regulating quorum sensing systems remains elusive (Lerat & Moran, 2004). However, an early bacterial ancestor has been suggested to have been a flagellated microbe with two membranes (Coleman et al., 2021). Extending such analyses about the origin of biofilm and biofilm‐related genes might provide novel insights into the treatment of biofilm infections (Kalia et al., 2019; Römling & Balsalobre, 2012).

In many bacterial species, an agar‐grown biofilm model has been identified (Römling et al., 1998; Shapiro, 1998). These colony morphotype biofilm models with dense association of individual cells embedded into a honey‐bee comb like extracellular matrix (Branda et al., 2001; Morris et al., 1996; Rice et al., 1992; Römling et al., 1998) can be seen in a light rather different than a laboratory curiosity resembling embryonic and tissue development in higher organisms (Asally et al., 2012; Chou et al., 2022; Futo et al., 2021). In fact, the dense association of microbial cells, a readily accessible and genetically screenable biofilm model, might not only be a physiologically and ecologically relevant model for biofilm formation in microbial‐dense environments such as the colon but also serve as a model for ancient environmental biofilms on surfaces (Boomer et al., 2009; MacKenzie et al., 2015; Ward et al., 1998). The subsequent free‐living planktonic life style is considered a temporary condition in the life cycle of most microbes (Futo et al., 2021; Henrici, 1933), which does, however, not contradict the multiple independent gain of nmulticellularity in eukaryotes (Parfrey & Lahr, 2013).

The fundamentally ancient process of energy conservation involving solid reversible redox active surfaces has many applications. One of the most prominent applications is the gain of energy in microbial fuel cells (Logan et al., 2006; Ucar et al., 2017). Another example is the gain of energy by electrosynthesis (Jourdin & Burdyny, 2021; Rabaey & Rozendal, 2010). In a reverse process, biofilms that protect steel from corrosion can be developed (Dubiel et al., 2002). Furthermore, the understanding of these ancient molecular mechanisms of biofilm formation might also aid to modulate biofilm formation (Maeda et al., 2007; Perona‐Vico et al., 2019) and tackle chronic infections (Nie et al., 2012; Soldano et al., 2021). Integrating the knowledge on these ancient processes of energy acquisition and its preservation in modern microbes might lead to innovative biofilm treatment strategies and new application processes.

## CONFLICT OF INTEREST

The author declares no conflict of interest.

## Supporting information


**Appendix S1** Supporting Information

## Data Availability

All data are available upon request.
