# Supplementary material for: Is biofilm formation intrinsic to the origin of life?
Source: Environ Microbiol. 2022 Sep 7;25(1):26–39. doi: 10.1111/1462-2920.16179 (PMC10086821; doi:10.1111/1462-2920.16179)
Supplement: Supplementary file 1 — Appendix S1 Supporting Information [file EMI-25-26-s001.docx]

Supplementary material

Is biofilm formation intrinsic to the origin of life?

Ute Römling

Department of Microbiology, Tumor and Cell Biology, Karolinska Institutet, SE-171 77 Stockholm, Sweden

ute.romling@ki.se; Tel.: 0046-8-52487319

Supplementary Material to Figure 3Aa and b:

BcsA_HYDRO (A0A432RG04, *Hydrogenothermus* sp.), CelA_SULAA (*Sulfurihydrogenibium azorense* Az-Ful), BcsA_AQUAE (O67406, *Aquifex aeolicus*), BcsA_SALTY ([Q93IN2](https://www.uniprot.org/uniprot/Q93IN2), *S. typhimurium*), BcsA_CLODIF (AJP12292.1, *Clostridioides difficile* 630), BcsA_CERSPH (*Cereibacter sphaeroides* ATCC 17023), GLYCOTRA_WOESEA (MBI4151113.1), GLYCOTRA_METHARC (CAG0966249.1), WP 013237893.1 (*Clostridium ljundahlii*), GLYCOTRA_PACEA (MBI5803293.1), WP 011306527.1 (*Methanosarcina barkeri* DSM804), WP 048119672.1 (*M. barkeri* 227), GLYCOTRA_METHAR (MBI4151113.1), PGAC_ECOLI (P75905, *E. coli* K-12), GLYCOTRA2_ACEAUT (BAL58984.1), ExoA_ACEAUT (BAL60196.1), GLYCOTRA_GT2_BIPANA (WP 122030831.1), WP 052712908.1 (*M. barkeri* 227), GLYCO_GT2_ACEAUT (BAL60260.1), GLYCOTRA1_BIPANA (WP 157959473.1), GLYCOTRA1_BIPSIB (QAA76168.1), GLYCOTRA3_ACEAUT (BAL59581.1), GLYCOTRA2_BIPSIB (QAA76636.1), GLYCOTRA2_BIPANA (WP 122030981.1).

Supplementary Material to Figure 3Ba:

GGDEF_ACEAUT (BAL59883.1); GGDEF1_BIPSIB (QAA76087.1); GGDEF2_BIPSIB (QAA76488.1); GGDEF3_ BIPSIB (QAA76751.1; GGDEF1_BPIANA (WP_122030618.1); GGDEF2_BIPANA (WP_122031498.1); GGDEF3_BIPANA (WP_162297725.1); GGDEF4_BIPANA (SQD92144.1).

Supplementary Material to Figure 3Ca:

CdaA_BACSU (Q45589, *Bacillus subtilis* 168); DacA_LISMO (Q8Y5E4, *Listeria monocytogenes* serovar 1/2a ATCC BAA-679); DacA_Parcu (A0A419G9B5, Candidatus *Parcubacteria* bacterium); DacA2_Parcu (NCO15553.1, Candidatus *Parcubacteria* bacterium); CdaM_MYCPN(A0A8D9FKA8, *Mycoplasma pneumoniae* ATCC 29342); DacB BACCR (Q812L9, *Bacillus cereus* ATCC 14579); CdaS_BACSU (O31854, *B. subtilis* 168); DisA_CLOST (WP_013240715.1, *Clostridium ljungdahlii* DSM 13528); DisA_BACSU (P37573, *B. subtilis* 168); DisA THEMA (Q9WY43, *T. maritima* ATCC 43589); DisA_ACEAU (BAL58991.1, Candidatus *A. autotrophicum*); DisA_BIPSI (QAA77389.1, Candidatus *B. sibiricus*); DisA_BIPAN (WP_122030516.1, Candidatus *B. anaerobius*); DACZ_METJA (Q58408, *Methanocaldococcus jannaschii* ATCC 43067); DacZ_METHJ (Q2FNI7, *Methanospirillum hungatei* JF-1 ATCC 27890); DacZ2_METHJ (Q2FM57, *Methanospirillum hungatei* JF-1); DacZ_METJA (Q58408, *Methanocaldococcus jannaschii* ATCC 43067).
